# The *E. coli* pET expression system revisited—mechanistic correlation between glucose and lactose uptake

**DOI:** 10.1007/s00253-016-7620-7

**Published:** 2016-05-27

**Authors:** David Johannes Wurm, Lukas Veiter, Sophia Ulonska, Britta Eggenreich, Christoph Herwig, Oliver Spadiut

**Affiliations:** 1 Research Division Biochemical Engineering, Institute of Chemical Engineering, Vienna University of Technology, Vienna, Austria; 2 Christian Doppler Laboratory for Mechanistic and Physiological Methods for Improved Bioprocesses, Institute of Chemical Engineering, Vienna University of Technology, Vienna, Austria

**Keywords:** *Escherichia coli* BL21(DE3), pET expression system, Lactose induction, Antibody fragment, Soluble protein, Mechanistic model

## Abstract

Therapeutic monoclonal antibodies are mainly produced in mammalian cells to date. However, unglycosylated antibody fragments can also be produced in the bacterium *Escherichia coli* which brings several advantages, like growth on cheap media and high productivity. One of the most popular *E. coli* strains for recombinant protein production is *E. coli* BL21(DE3) which is usually used in combination with the pET expression system. However, it is well known that induction by isopropyl β-d-1-thiogalactopyranoside (IPTG) stresses the cells and can lead to the formation of insoluble inclusion bodies. In this study, we revisited the pET expression system for the production of a novel antibody single-chain variable fragment (scFv) with the goal of maximizing the amount of soluble product. Thus, we (1) investigated whether lactose favors the recombinant production of soluble scFv compared to IPTG, (2) investigated whether the formation of soluble product can be influenced by the specific glucose uptake rate (*q*_s,glu_) during lactose induction, and (3) determined the mechanistic correlation between the specific lactose uptake rate (*q*_s,lac_) and *q*_s,glu_. We found that lactose induction gave a much greater amount of soluble scFv compared to IPTG, even when the growth rate was increased. Furthermore, we showed that the production of soluble protein could be tuned by varying *q*_s,glu_ during lactose induction. Finally, we established a simple model describing the mechanistic correlation between *q*_s,lac_ and *q*_s,glu_ allowing tailored feeding and prevention of sugar accumulation. We believe that this mechanistic model might serve as platform knowledge for *E. coli*.

**Electronic supplementary material:**

The online version of this article (doi:10.1007/s00253-016-7620-7) contains supplementary material, which is available to authorized users.

## Introduction

Antibodies are used to treat a wide variety of human diseases. More than 35 monoclonal antibodies and antibody fragments have been commercialized, and around 240 therapeutic monoclonal antibodies and antibody fragments are in clinical trials (Lee and Jeong 2015). Since more than 1000 kg of these therapeutics are needed per year worldwide, there is an urge for cheap and fast production (Elvin et al. 2013; Lee and Jeong 2015; Liu 2014; Rodrigues et al. 2010; Walsh 2014). Due to the requirement of post-translational modifications, most therapeutic monoclonal antibodies and antibody fragments are produced in mammalian cells to date. However, there are many drawbacks such as glycan heterogeneity, low volumetric productivity, long cultivation times, expensive media, and the potential risk of virus contamination (Khan 2013; Lee and Jeong 2015). Thus, the prokaryotic organism *Escherichia coli* has been investigated as alternative host for the production of unglycosylated antibody fragments, mainly single-chain variable fragments (scFv), which are also suitable for antigen detection (Lee and Jeong 2015; Spadiut et al. 2014; Wals and Ovaa 2014). *E. coli* can be cultivated on inexpensive media to high cell densities and has a high growth rate; its genetics are very well characterized and an increasingly large number of cloning vectors and mutant host strains are available (e.g., Baeshen et al. 2015; Rosano and Ceccarelli 2014). The *E. coli* strain BL21(DE3) and its derivatives are by far the most used *E. coli* strains for recombinant protein production as they exhibit several biotechnological advantages compared to other *E. coli* strains, such as low acetate yield, high biomass yield, and reduced expression of proteases (Choi et al. 2006; Ferrer-Miralles et al. 2015; Rosano and Ceccarelli 2014). Usually, the well-known pET expression system is used in combination with *E. coli* BL21(DE3) (Studier and Moffatt 1986). The lac operon can be induced by allolactose and its molecular mimic isopropyl β-d-1-thiogalactopyranoside (IPTG) (Neubauer et al. 1992). IPTG is a very strong inducer that is not metabolized by *E. coli*, which is why one point addition is sufficient. Thus, IPTG is usually used in industrial production processes with *E. coli* BL21(DE3). However, IPTG is known to put a high metabolic burden on the cells resulting in the formation of inactive aggregates of the recombinant target protein, known as inclusion bodies (IBs). Thus, lactose has been studied as alternative inducer. Lactose was found to be as effective as IPTG, to increase cell fitness, to reduce IB formation, and to enhance the formation of soluble recombinant product (Bashir et al. 2015; Fruchtl et al. 2015; Gombert and Kilikian 1998; Neubauer et al. 1992; Pei et al. 2011; Zou et al. 2014). However, lactose is metabolized by *E. coli* making stable induction more complicated as it has to be continuously supplied (Striedner et al. 2003). In a previous study, it was nicely shown that lactose metabolism strongly depends on the available amount of glucose (Kremling et al. 2015). However, a potential mechanistic correlation between glucose and lactose uptake has not been investigated yet.

In this study, we used *E. coli* BL21(DE3) and the pET expression system for the production of a novel scFv (Stadlmann et al. 2015). We hypothesized that induction by lactose increases the amount of soluble product compared to IPTG. Thus, we (1) tested and compared IPTG and lactose as inducer, (2) investigated whether the formation of soluble product can be influenced by the specific uptake rate of glucose during induction with lactose, and (3) determined a mechanistic correlation between the specific uptake rates of lactose and glucose.

## Materials and methods

### Strain

*E. coli* BL21(DE3) (Life technologies, Carlsbad, CA, USA) and the pET28a(+) expression system were used for production of the recombinant scFv which describes an engineered IgY fragment against PT-gliadin useful for the treatment of celiac disease (Stadlmann et al. 2015).

### Bioreactor cultivations

#### Media

A defined minimal medium according to DeLisa et al. (1999) supplemented with 0.02 g/L kanamycin and different amounts of glucose and lactose (Table 1) was used for all cultivations.

**Table 1:** Sugar concentrations in different DeLisa media

Component	Pre-culture	Batch	Feed glucose	Feed lactose
C_6_H_12_O_6_·H_2_O (g/L)	8.8	22.0	275	–
C_12_H_22_O_11_·H_2_O (g/L)	–	–	–	210

#### Pre-culture

A 500-mL sterile DeLisa pre-culture medium were inoculated from frozen stocks (1.5 mL, −80 °C) and incubated in a 2500-mL High-Yield shake flask in an Infors HR Multitron shaker (Infors, Bottmingen, Switzerland) at 37 °C and 230 rpm for 20 h. Then a 4500-mL DeLisa-batch medium in the bioreactor was inoculated with 500 mL of pre-culture.

#### Batch and fed-batch cultivations

Batch and fed-batch cultivations were done in a stainless steel Satorius Biostat Cplus bioreactor (Satorius, Göttingen, Germany) with a working volume of 10 L. The bioreactor was aerated with a mixture of pressurized air and pure oxygen at 1.5 vvm and agitated constantly at 1000 rpm. Dissolved oxygen (dO_2_) was monitored with a fluorescence dissolved oxygen electrode Visiferm DO425 (Hamilton, Reno, NV, USA) and kept above 40 % throughout all cultivations by varying the ratio of pressurized air to pure oxygen. pH was monitored with an EasyFerm electrode (Hamilton, Reno, NV, USA) and maintained constant at pH 7.2 by addition of NH_4_OH (12.5 %). Base consumption was determined gravimetrically. CO_2_ and O_2_ concentrations in the off-gas were monitored by a DASGIP GA gas analyzer (Eppendorf, Hamburg, Germany). All process parameters were adjusted and logged by the process information management system Lucullus (Biospectra, Schlieren, Switzerland).

The batch phase was conducted at 35 °C and yielded a biomass concentration of 8–9 g dry cell weight (DCW) per liter. After depletion of glucose, visible by a drop in the CO_2_ off-gas signal, a fed-batch to generate a biomass was conducted. We fed at a constant specific glucose uptake rate (*q*_s,glu_) of 0.2 g/g/h. When the final DCW reached 25 g/L, the culture was induced by IPTG or lactose, respectively. During non-induced fed-batch and induction with IPTG, DCW in the bioreactor was estimated using a soft-sensor tool (Wechselberger et al. 2013). During induction with lactose, DCW was calculated assuming a constant biomass yield (*Y*_*X*/*S*_ = 0.37 g/g, own unpublished data). The feed rate was adjusted to maintain a constant *q*_s,glu_ and was calculated according to Eq. 1.

1 $$ F={q}_{\mathrm{s}}\cdot X\cdot \frac{V}{W} $$

*F*: Feed rate (g/h)
*q*_s_: Specific substrate uptake rate (g/g/h)
*X*: DCW concentration (g/L)
*V*: Reactor volume (L)
*W*: Amount of substrate per feed (g/g)

#### Cultivation strategy

In this study, cultivations following a standard procedure comprising three phases (batch, non-induced fed-batch, induced fed-batch) as well as dynamic experiments (pulses and shifts) were carried out. Applying dynamic process conditions to accelerate strain characterization and bioprocess development is a common approach in our working group (Dietzsch et al. 2011a, 2011b; Jazini and Herwig 2011; Spadiut et al. 2013; Zalai et al. 2012). An overview of the different cultivations and their respective goals is shown in Supplementary Table S1. Induction was either performed by 0.5 mM IPTG or by lactose which was applied either as pulses or by continuous feeding. In these cultivations, the lactose concentration in the media was kept in excess between 5 and 15 g/L.

#### Sampling

Samples were taken at the beginning and the end of the batch and the non-induced fed-batch. During induction, sampling was performed at the beginning and the end of each shift/pulse and every hour during fed-batch cultivations. Specific product formation rates and final product yields are given for an induction phase of approximately 4 h for all cultivations. DCW was determined by centrifugation (4500*g*, 4 °C, 10 min) of 5 mL cultivation broth, washing the obtained cell pellet with a 0.1 % NaCl solution and subsequent drying at 105 °C for 48 h. Optical density at 600 nm (OD_600_) was measured in the photometer Genesys 20 (Thermo Scientific, Waltham, MA, USA). Samples were diluted with deionized water to stay within the linear range of the photometer (OD_600_ 0.1–0.8). A linear correlation between OD_600_ and DCW was established to verify and, if necessary, correct the DCW estimation of the soft-sensor (Eq. 2).

2 $$ DCW={OD}_{600}\cdot 0.445 $$

DCW: Biomass dry cell weight (g/L)
OD_600_: Optical density at 600 nm

### Substrate and metabolite quantification

Cell-free samples of the cultivation broth were analyzed for concentrations of substrates and metabolites by HPLC (Agilent Technologies, Santa Clara, CA, USA) with a Supelcogel C-610 H ion exchange column (Sigma-Aldrich, St. Louis, MO, USA) and a refractive index detector (Agilent Technologies, Santa Clara, CA, USA). The mobile phase was 0.1 % H_3_PO_4_ with a constant flow rate of 0.5 mL/min, and the system was run isocratically at 30 °C.

### Product quantification

Cells were harvested (4500*g*, 4 °C, 10 min), resuspended, and diluted in Tris buffer (100 mM, 10 mM EDTA, pH 7.4) to a DCW concentration of 5 g/L and subsequently homogenized in an EmusiflexC3 Homgeniziser (Avestin, Ottowa, ON, USA) at 1500 bar for five passages. After centrifugation (14,000*g*, 4 °C, 10 min), soluble protein (SP) was recovered in the supernatant and IBs in the pellet.

Cell debris from 0.5 mg DCW was resuspended in 1× Laemmli buffer, and the supernatant was diluted with 2× Laemmli buffer before the samples were heated at 95 °C for 10 min. Ten microliters of each sample were loaded onto pre-cast SDS gels (8–16 %) (GE Healthcare, Little Chalfont, UK). Gels were run in an Amersham ECL Gel Box, a horizontal mini-gel system (GE Healthcare, Little Chalfont, UK) for 90 min at 140 V and stained with Coomassie Blue. On every gel, three BSA standards (0.5 μg, 1.5 μg, 3 μg per lane) were applied. The protein bands were evaluated densitometrically using the software Image Lab (Bio-Rad, Hercules, CA, USA). Calibrations always gave an *R*^2^ above 0.96 and allowed the quantification of SP and IBs. This method has been used in over 700 publications to date and is known to give precise data (e.g., Matusica et al. 2016; Wahlang et al. 2016; Xu et al. 2016).

## Results

In this study, we revisited the pET expression system in *E. coli* BL21(DE3) for the production of a novel scFv. We wanted to (1) prove that lactose favors the recombinant production of soluble scFv compared to IPTG, (2) investigate if the formation of soluble product can be influenced by *q*_s,glu_ during lactose induction, and (3) determine a mechanistic correlation between *q*_s,lac_ and *q*_s,glu_.

### Induction by IPTG vs. lactose

**Fig. 1:**
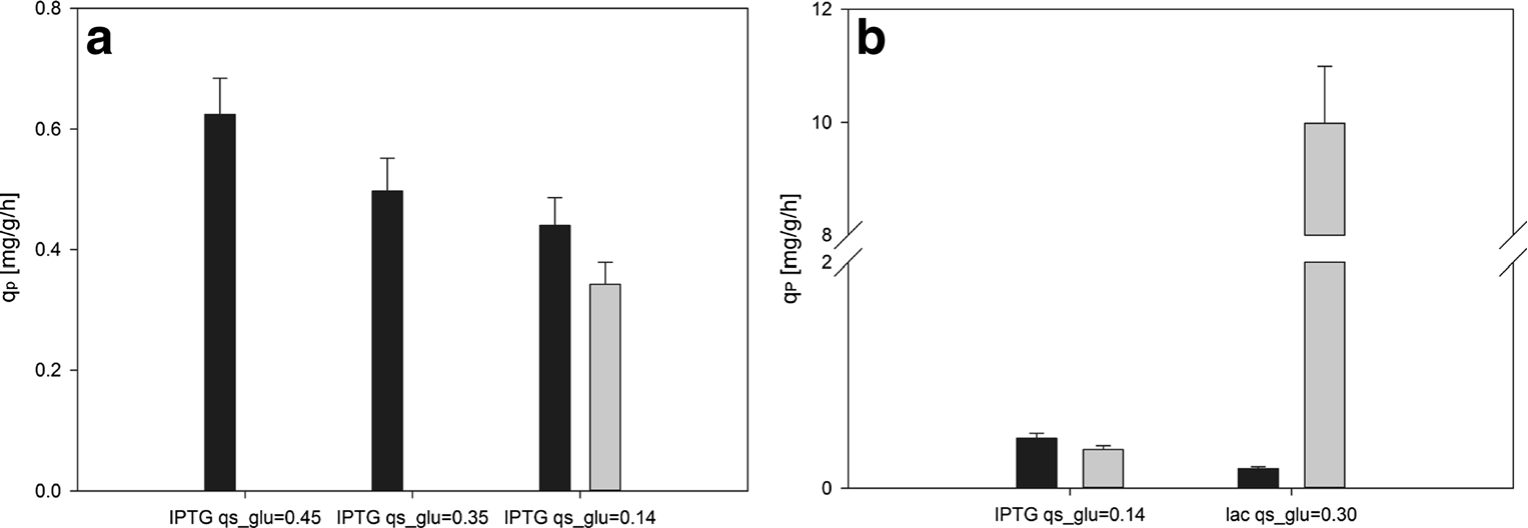
**a** Specific product formation rate (*q*_P_) at different *q*_s,glu_ during IPTG induction. *Black bars* indicate specific production rate of inclusion bodies (*q*_P,IB_), and *gray bars* specific production rate of soluble product (*q*_P,SP_). **b** Specific product formation rate (*q*_P_) at different *q*_s,glu_ during IPTG or lactose induction. *Black bars* indicate specific production rate of inclusion bodies (*q*_P,IB_), and *gray bars* specific production rate of soluble product (*q*_P,SP_)

We tested and compared IPTG and lactose as inducers for the pET expression system. Slow uptake of glucose and thus a low specific growth rate (*μ*) was described to result in higher amount of SP (Shin et al. 1998). Thus, we wanted to investigate whether this strategy was also suitable for the expression of the novel scFv and conducted three fed-batch cultivations with different *q*_s,glu_ during induction with 0.5 mM IPTG (Fig. 1a, Table 2).

**Table 2:** Strain physiological parameters of *E. coli* BL21(DE3) producing a scFv via a pET expression system during either IPTG or lactose induction

Inducer	*q*_s,glu_ (g/g/h)	*μ* (1/h)	*q*_P,IB_ (mg/g/h)	Std. dev (mg/g/h)	*q*_P,SP_ (mg/g/h)	Std. dev (mg/g/h)	*q*_P,acetate_ (mg/g/h)	Std. dev (mg/g/h)	IB-titer (mg/g)	SP-titer (mg/g)
IPTG	0.45	0.16	0.62	0.059	0.00		3.11	0.006	2.50	0.00
IPTG	0.35	0.08	0.50	0.055	0.00		2.65	0.005	1.99	0.00
IPTG	0.14	0.03	0.44	0.046	0.34	0.036	0.24	0.001	1.76	1.37
Lactose	0.30	0.10	0.17	0.021	9.99	0.899	0.00		0.69	39.55

At *q*_s,glu_ of 0.45 g/g/h and 0.35 g/g/h only IBs were formed during IPTG induction. IB formation decreased when feeding at lower *q*_s,glu_. At *q*_s,glu_ of 0.14 g/g/h, soluble scFv was produced. Apparently the amount of SP and IBs was strongly linked to *q*_s,glu_ and thus *μ*. Furthermore, by decreasing the feeding rate from *q*_s,glu_ 0.45 to 0.14 g/g/h, the acetate formation rate was reduced more than 10-fold from 3.11 to 0.24 mg/g/h. It is known that the formation of acetate has a negative impact on cell growth and recombinant protein production and should thus be kept at a minimum to ensure high product quality (De Mey et al. 2007). Our findings that a low *μ* leads to higher production of SP as well as lower acetate formation are in good agreement with literature (Hellmuth et al. 1994; Sanden et al. 2005; Shin et al. 1998). Nevertheless, more than half of the scFv was still found as insoluble and inactive IBs. A further decrease of *q*_s,glu_ to potentially increase the amount of SP was not feasible as it would have led to slow growth and thus very long process times. Therefore, the alternative inducer lactose which is described to enhance cell fitness and to increase the amount of SP (Donovan et al. 1996) was tested (Fig. 1b, Table 2). Even though *q*_s,glu_ was 0.30 g/g/h during lactose induction and thus more than 2-fold higher than in the only experiment with IPTG which gave SP (*q*_s,glu_ = 0.14 g/g/h) around 30-fold more soluble scFv was produced. Furthermore, the acetate concentration was below the detection limit when lactose was used as inducer (Table 2).

### Tunability of recombinant protein production by varying *q*_s,glu_ during lactose induction

**Fig. 2:**
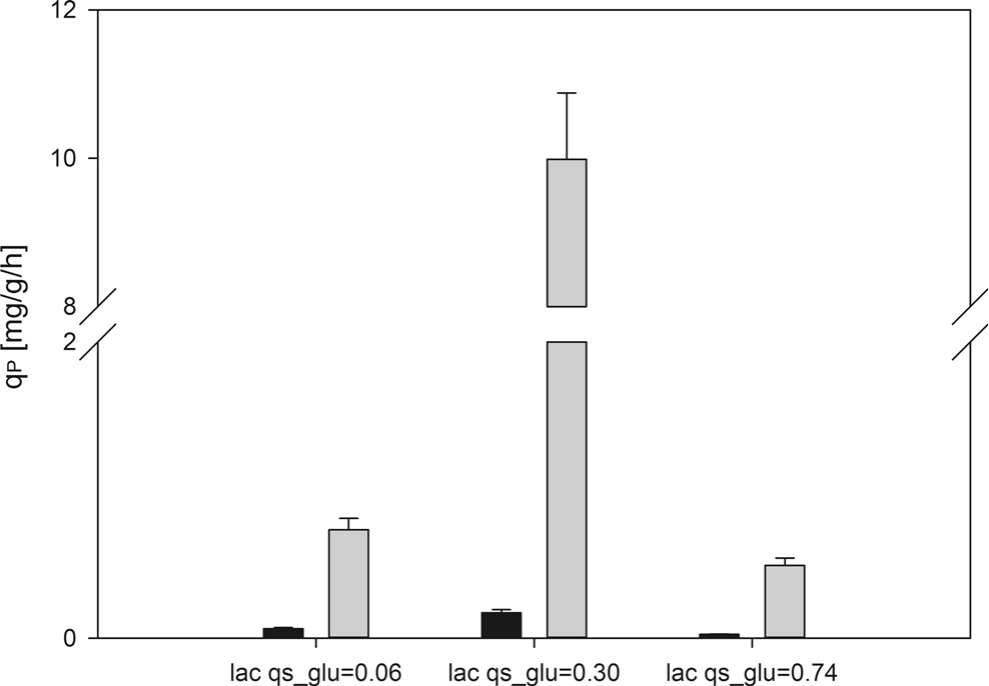
Specific product formation rate (*q*_P_) at different *q*_s,glu_ during lactose induction. *Black bars* indicate specific production rate of inclusion bodies (*q*_P,IB_), and *gray bars* specific production rate of soluble product (*q*_P,SP_)

Furthermore, we investigated the potential tunability of recombinant protein production by testing three different *q*_s,glu_ during induction with lactose, which was always present in excess (Fig. 2).

Figure 2 shows that both the formation of SP and IBs strongly depended on *q*_s,glu_ during lactose induction. There was a clear optimum at *q*_s,glu_ = 0.30 g/g/h. At *q*_s,gluc_ of 0.06 g/g/h 14-fold less SP was produced and also IB formation was reduced 3-fold. We speculate that at this low *q*_s,glu_, the cells basically only had enough energy for maintenance metabolism but not for recombinant protein production. However, we were rather surprised to see reduced production of both SP and IB also at the higher *q*_s,glu_ of 0.74 g/g/h. Thus, we analyzed *q*_s,lac_ at the respective *q*_s,glu_. As shown in Table 3, *q*_s,lac_ was strongly dependent on *q*_s,glu_. At *q*_s,glu_ of 0.74 g/g/h, only 0.02 g/g/h lactose were metabolized. We speculate that this amount of inducer was too low to guarantee full induction, thus resulting in reduced productivity.

**Table 3:** Strain physiological parameters of *E. coli* BL21(DE3) producing a scFv via a pET expression system during lactose induction at different *q*_s,glu_

Inducer	*q*_s,glu_ (g/g/h)	*q*_s,lac_ (g/g/h)	*q*_P,IB_ (mg/g/h)	Std. dev (mg/g/h)	*q*_P,SP_ (mg/g/h)	Std. dev. (mg/g/h)	*q*_P,acetate_ (mg/g/h)	IB-titer (mg/g)	SP-titer (mg/g)
Lactose	0.06	0.09	0.06	0.007	0.73	0.077	0.00	0.26	2.92
	0.30	0.08	0.17	0.021	9.99	0.899	0.00	0.69	39.55
	0.74	0.02	0.03	0.003	0.49	0.049	0.00	0.12	1.96

### Mechanistic correlation between *q*_s,lac_ and *q*_s,glu_

Motivated by our observation of an apparent mechanistic correlation between *q*_s,lac_ and *q*_s,glu_ (Table 3), we performed several cultivations to shed more light on this physiological correlation. First, we performed a batch cultivation with excess of both glucose and lactose. In this experiment, we observed the well-known phenomenon of carbon catabolite repression (Stulke and Hillen 1999) meaning that as long as glucose was present in excess, no lactose was taken up (Supplementary Fig. S1). Only when glucose was depleted, lactose was metabolized at a very slow rate of 0.05 g/g/h, which is in agreement to literature (Kremling et al. 2015).

**Fig. 3:**
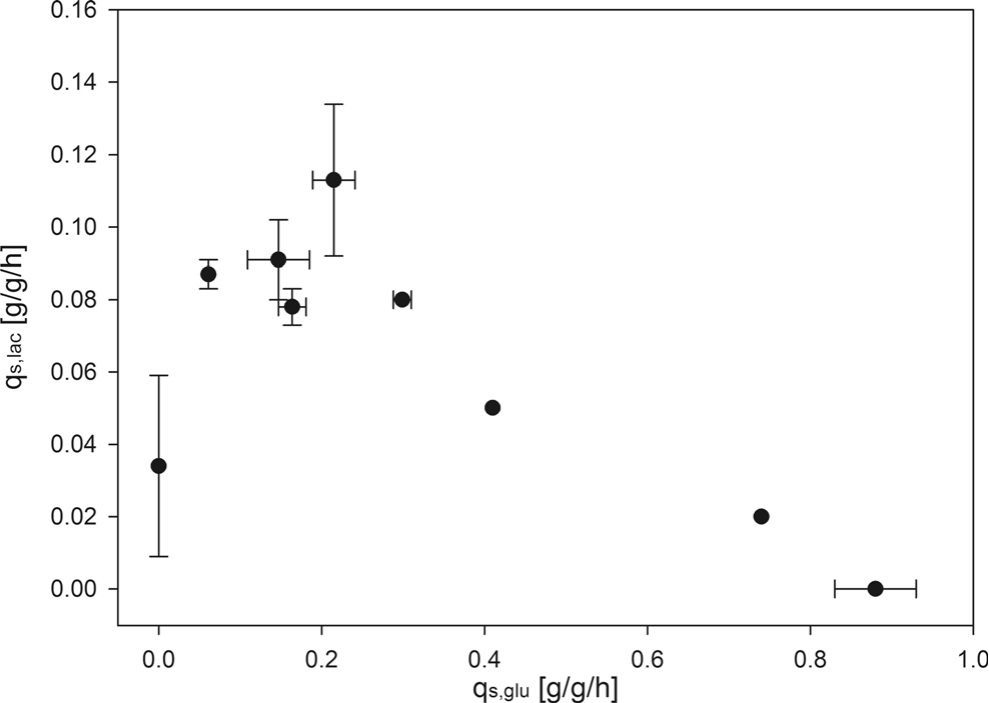
*q*_s,lac_ as a function of *q*_s,glu_ for the recombinant *E. coli* strain producing scFv with the pET expression system

On the contrary, at limiting amounts of glucose but an excess of lactose, we observed a much higher *q*_s,lac_ (Table 3). Several subsequent cultivations revealed that *q*_s,lac_ was in fact a function of *q*_s,glu_ (Fig. 3, Table 4).

**Table 4:** Experimentally evaluated *q*_s,lac_ at respective *q*_s,glu_ for the recombinant *E. coli* strain producing scFv with the pET expression system

*q*_s,glu_ (g/g/h)	Std. dev (g/g/h)	*q*_s,lac_ (g/g/h)	Std. dev (g/g/h)
0.00	–	0.03	0.025
0.06	–	0.09	0.004
0.15	0.038	0.09	0.011
0.16	0.017	0.08	0.005
0.22	0.026	0.11	0.021
0.30	0.011	0.08	–
0.41	–	0.05	–
0.74	–	0.02	–
0.88	0.050	0.00	–

As shown in Fig. 3, *q*_s,lac_ increased with increasing *q*_s,glu_ having a maximum at *q*_s,glu_ of 0.2–0.25 g/g/h, before it decreased again. We used these findings to generate a simple mechanistic model describing *q*_s,lac_ as a function of *q*_s,glu_. Such a mechanistic model would greatly facilitate bioprocess development as only a few experiments would be required to determine the correlation between *q*_s,lac_ and *q*_s,glu_. Thus, lactose accumulation and resulting osmotic stress can be reduced (Shiloach and Fass 2005) and tailored feeding and induction are possible. We hypothesized that the correlation between *q*_s,lac_ and *q*_s,glu_ was described by two phenomena, namely (1) *q*_s,lac_ depended Monod-like on *q*_s,glu_ until a certain maximum was reached, before (2) *q*_s,lac_ decreased at high *q*_s,glu_ which was treated similarly to the phenomenon of substrate inhibition (Sivakumar et al. 1994). To describe this correlation, we adapted the model proposed by Han and Levenspiel (1988) (Eq. 3).

3 $$ {q}_{\mathrm{s},\mathrm{lac}}={q}_{\mathrm{s},\mathrm{lac}, \max}\cdot \max \left({\left(1-\frac{q_{\mathrm{s},glu}}{q_{\mathrm{s},glu,\mathrm{crit}}}\ \right)}^n,0\right)\cdot \left(\frac{q_{\mathrm{s},glu}}{q_{\mathrm{s},glu}+{K}_{\mathrm{A}}{\left(1-\frac{q_{\mathrm{s},glu}}{q_{\mathrm{s},glu,\mathrm{crit}}}\right)}^m}+\frac{q_{\mathrm{s},\mathrm{lac},\mathrm{noglu}}}{q_{\mathrm{s},\mathrm{lac}, \max }}\ \right) $$

*q*_s,lac_: Specific lactose uptake rate (g/g/h)
*q*_s,lac,max_: Maximum specific lactose uptake rate (g/g/h)
*q*_s,glu_: Specific glucose uptake rate (g/g/h)
*q*_s,glu,crit_: Critical specific glucose uptake rate up to which lactose is consumed (g/g/h)
*q*_s,lac,noglu_: Specific lactose uptake rate at *q*_s,glu_ = 0 (g/g/h)
*K*_A_: Affinity constant for the specific lactose uptake rate (g/g/h)
*m*, *n*: Type of inhibition (noncompetitive, uncompetitive, competitive)

The unknown parameters in this model are the maximum specific lactose uptake rate (*q*_s,lac,max_), the critical specific glucose uptake rate up to which lactose is consumed (*q*_s,glu,crit_), the affinity constant (*K*_A_), the two constants *n* and *m* indicating the type of inhibition (noncompetitive, uncompetitive, competitive; (Han and Levenspiel 1988)) and the specific lactose uptake rate at zero glucose uptake (*q*_s,lac,noglu_). For the parameter identification, the Nelder-Mead simplex method in MATLAB (Lagarias et al. 1998) was used to minimize the objective function (Eq. 4) which describes the distance between the experimental data and the predicted values by the model. For points, where no standard deviation was available, the mean of all standard deviations was taken.

4 $$ S={\displaystyle \sum_{i=1}^n}{\left(\ \frac{q_{\mathrm{s},\mathrm{lac}, \max, \mathrm{meas},\mathrm{i}}-{q}_{\mathrm{s},\mathrm{lac}, \max, \mathrm{model},\mathrm{i}}}{\sigma_i}\right)}^2 $$

*S*: objective function
*q*_s,lac,meas,i_: *i*th measurement of *q*_s,lac_
*q*_s,lac,model,i_: predicted *q*_s,lac_ at timepoint of *i*th measurement
*σ*_*i*_: standard deviation of the *i*th data point

Based on our observations, we defined that *q*_s,lac_ is greater than zero when no glucose is consumed. Furthermore, parameter values must have a mechanistic meaning, which is why we assumed *q*_s,lac_, *K*_A_, and *n* to be positive and even constrained them further (e.g., *q*_s,lac_ < *q*_s,glu,crit_, *K*_A_ < 1, *n* > 0, etc.). To analyze the model and to evaluate the impact of the model parameters, we performed a sensitivity analysis, where we increased or decreased the parameters by 20 % (an example is shown in Supplementary Fig. S2).

The sensitivity analysis revealed the parameter *m* to have almost no impact on the curve. Furthermore, the parameter estimation revealed that this parameter is almost zero (6.2 × 10^−9^). This is in accordance with our assumption of a noncompetitive inhibition which is described by *m* = 0 (Han and Levenspiel 1988). Thus, we set *m* to zero and simplified the model (Eq. 5).

5 $$ {q}_{\mathrm{s},\mathrm{lac}}={q}_{\mathrm{s},\mathrm{lac}, \max}\cdot \max \left({\left(1-\frac{q_{\mathrm{s},glu}}{q_{\mathrm{s},glu,\mathrm{crit}}}\ \right)}^n,0\right)\cdot \left(\frac{q_{\mathrm{s},glu}}{q_{\mathrm{s},glu}+{K}_{\mathrm{A}}}+\frac{q_{\mathrm{s},\mathrm{lac},\mathrm{noglu}}}{q_{\mathrm{s},\mathrm{lac}, \max }}\ \right) $$

**Fig. 4:**
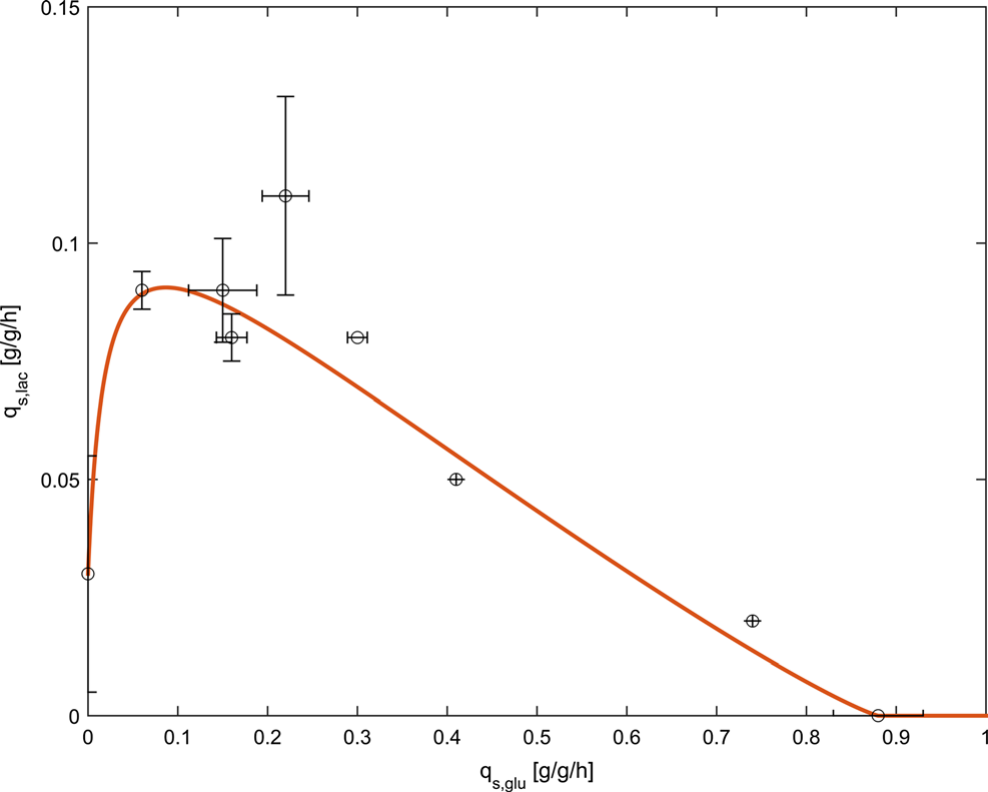
Optimal fit with parameters *q*_s,lac,max_ = 0.088 g/g/h, *q*_s,glu,crit_ = 0.88 g/g/h, *q*_s,lac,noglu_ = 0.034 g/g/h, *K*_A_ = 0.019 g/g/h, *n* = 1.16

Additionally, the sensitivity analysis showed that *n*, *q*_s,lac,max_, *K*_A_, *q*_s,lac,noglu_, and *q*_s,glu,crit_ had a significant impact on the curve. However, the impact of *n* was rather small and even a potential error of 20 % would lead to only small deviations of the curve (Supplementary Fig. S2). We used our experimental data for the recombinant *E. coli* strain producing the novel scFv and fitted the mechanistic model to the data using Eq. 5. The curve with the estimated parameters fitted the data with a normalized root mean square error (NRMSE) of 10.3 % and a coefficient of variation (CV) of 18.6 %. Furthermore, all parameters showed physiologically reasonable values. The resulting curve and the corresponding parameter values are shown in Fig. 4 and Table 5, respectively.

**Table 5:** Parameters of optimal fit for the mechanistic correlation between q_s,lac_ and *q*_s,glu_ for the recombinant *E. coli* strain producing scFv with the pET expression system

*q*_s,lac,max_ (g/g/h)	*q*_s,glu,crit_ (g/g/h)	*q*_s,lac,noglu_ (g/g/h)	*K*_A_ (g/g/h)	*n* (−)
0.088	0.88	0.034	0.019	1.16

## Discussion

In this study, we used a pET expression system and an *E. coli* BL21(DE3) strain for the production of a novel antibody single-chain variable fragment. Since we wanted to maximize the amount of soluble product and reduce the formation of IBs, we (1) analyzed whether lactose favors the recombinant production of soluble scFv compared to IPTG, (2) investigated whether the formation of soluble product can be influenced by *q*_s,glu_ during lactose induction, and (3) determined the mechanistic correlation between *q*_s,lac_ and *q*_s,glu_.

In fact, we showed that lactose allowed a much higher production of soluble product compared to IPTG even when *μ* was increased. This outcome is in good agreement not only with several recent studies that also showed the benefits of using lactose as inducer compared to IPTG for other recombinant products (Bashir et al. 2015; Fruchtl et al. 2015; Zou et al. 2014) but also for antibody fragments (Donovan et al. 2000; Sanchez-Arreola et al. 2013). However, data on potential tunability of recombinant protein production by changing *q*_s,glu_ and thus *μ* during lactose induction are scarce to date.

Thus, we investigated a potential tunability of recombinant protein production and found that the formation of soluble product could be tuned by varying *q*_s,glu_ during lactose induction. Furthermore, we observed a mechanistic correlation between *q*_s,glu_ and *q*_s,lac_ in these experiments which motivated us to analyze this phenomenon in more detail.

In fact, we determined a mechanistic correlation between *q*_s,lac_ and *q*_s,glu_ and established a simple model. The shape of this curve can be explained by different phenomena. At high *q*_s,glu_, no lactose is taken up which can be explained by the phenomenon called carbon catabolite repression. When *q*_s,glu_ decreases, cAMP gets formed inside the cell. cAMP binds to the catabolite activator protein (CAP) which undergoes a change in conformation and binds to the promoter region of the *lac* operon. Consequently, transcription of the *lac* operon’s genes involving lactose permease and β-galactosidase is initiated and lactose can be taken up. However, in the absence of glucose, there is hardly any lactose taken up. The transport of lactose into the cell is ATP related (Voet et al. 2010). Without glucose metabolism, basically no ATP is generated, which is why this transport can barely happen (Kremling et al. 2015; Luo et al. 2014; Mayer et al. 2014; Siegal 2015).

The outcomes of this study can be used for strain characterization and fast bioprocess development. The values for the parameters *q*_s,lac,noglu_ and *q*_s,glu,crit_, which have a high impact on the curve, can be easily determined by simple batch cultivations. To determine the other parameters (*q*_s,lac,max_, *K*_A_, and *n*), we recommend performing two to three experiments where lactose is provided in excess and *q*_s,glu_ is adjusted to values below *q*_s,glu,crit_. In the optimal case, one of these *q*_s,glu_ values corresponds to the maximum of *q*_s,lac_. However, if this is not the case, *q*_s,lac,max_ is simply underestimated, leaving no risk for lactose accumulation. By performing these few experiments, enough data can be gathered to establish the mechanistic model. The mechanistic model can then be used to interpolate unknown *q*_s,lac_ values to *q*_s,glu_ points and thus allows fed-batch fermentations at different *q*_s,glu_ preventing unwanted lactose accumulation. Furthermore, *q*_s,lac_ can be adjusted within the feasible range allowing to tune the production of soluble protein, as we showed for the novel scFv (Table 3). By this approach, the production of soluble and active protein can be significantly increased.

Summarizing this work emphasizes the applicability of lactose, a cheap, nontoxic waste product, as inducer for the production of soluble recombinant proteins in *E. coli.* In future studies, we will investigate if the mechanistic model established in this study describes platform knowledge applicable to different *E. coli* strains. We suggest that by performing two-batch experiments and two to three fed-batches at different *q*_s,glu_ and concomitant lactose excess, enough data are available to fit the model and obtain the mechanistic correlation for basically any *E. coli* strain. Furthermore, we will test if induction by lactose triggers expression on a cellular level or if *E. coli* subpopulations are generated.

## Electronic supplementary material

ESM 1 (PDF 7255 kb)
