# Supplementary material for: The E. coli pET expression system revisited—mechanistic correlation between glucose and lactose uptake
Source: Appl Microbiol Biotechnol. 2016 May 27;100(20):8721–9. doi: 10.1007/s00253-016-7620-7 (PMC5035661; doi:10.1007/s00253-016-7620-7)
Supplement: Supplementary file 1 — (PDF 7255 kb) [file 253_2016_7620_MOESM1_ESM.pdf]

Applied Microbiology and Biotechnology

Supplementary Material for

Title: The *E. coli* pET expression system revisited – mechanistic correlation between glucose and lactose uptake

David Wurm[1], Lukas Veiter[1], Sophia Ulonska[1], Britta Eggenreich[1,2], Christoph Herwig[1,2], Oliver Spadiut[1,2,§]

[1]Research Division Biochemical Engineering, Institute of Chemical Engineering, Vienna University of Technology, Vienna, Austria

[2] Christian Doppler Laboratory for Mechanistic and Physiological Methods for Improved Bioprocesses, Institute of Chemical Engineering, Vienna University of Technology, Vienna, Austria

[§] Corresponding author: Oliver Spadiut, Vienna University of Technology, Institute of Chemical Engineering, Research Area Biochemical Engineering, Gumpendorfer Strasse 1a, 1060 Vienna, Austria. Tel: +43 1 58801 166473, Fax: +43 1 58801 166980, Email: oliver.spadiut@tuwien.ac.at

Supplementary Table S1 Overview of cultivations conducted in this study to evaluate $q_{s,lac}$ at a certain $q_{s,glu}$ and to investigate impact on product formation

**Cultivation**	**Inducer**	**Induction conditions**	**$q_{s,glu}$ [g/g/h]**	**Goal**
$B_{lac}1$	lactose	glucose and lactose in excess	$q_{s,max}$	Evaluate $q_{s,lac}$
$B_{lac}2$	lactose	no glucose, lactose in excess	0	Evaluate $q_{s,lac}$
$FB_{IPTG}1$ - 3	IPTG	0.5 mM IPTG, constant $q_{s,glu}$	0.14-0.45	Impact on q_p
$FB_{lac}1$-3	lactose	lactose in excess,<br>glucose via pulse or feeding	0.05-0.88	Impact on q_p, evaluate $q_{s,lac}$,

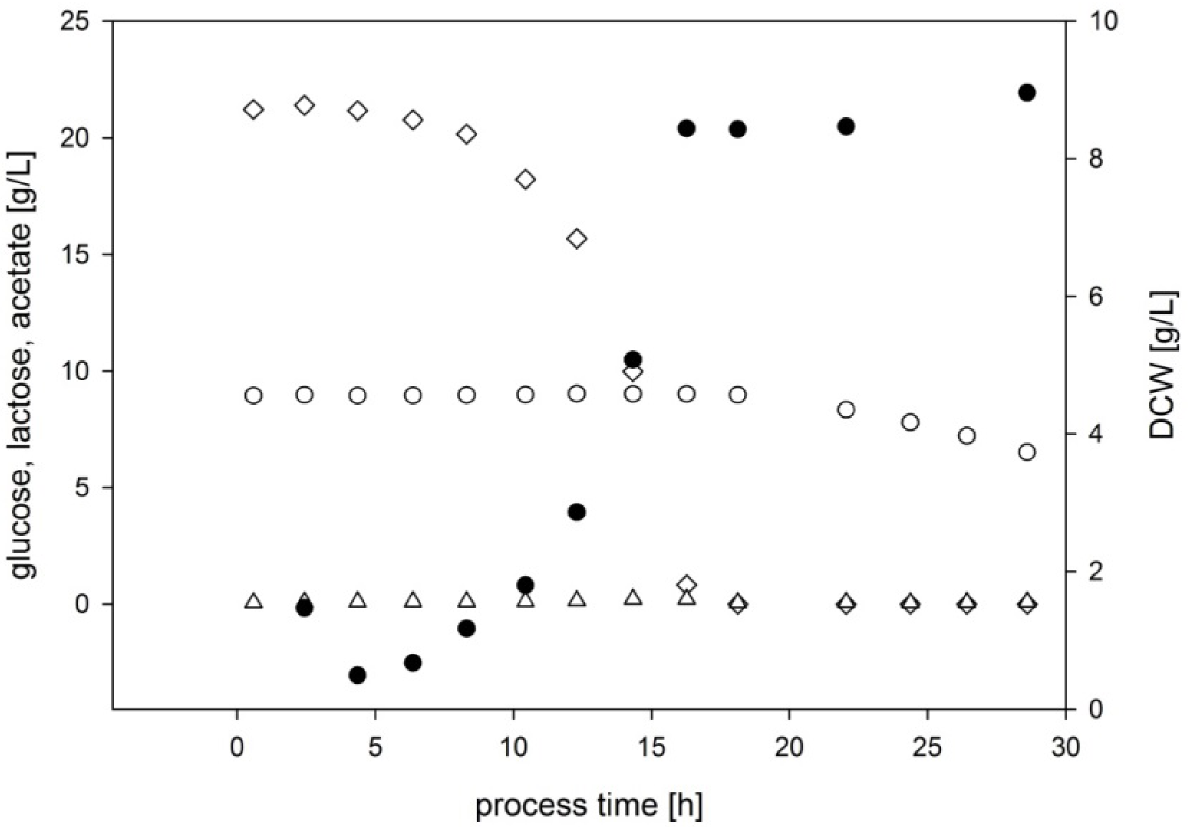

Supplementary Fig. S1 Batch cultivation in the presence of both glucose and lactose in excess. Carbon catabolite repression is nicely shown. Black dots, DCW; empty diamonds, glucose; empty circles, lactose; empty triangles, acetate

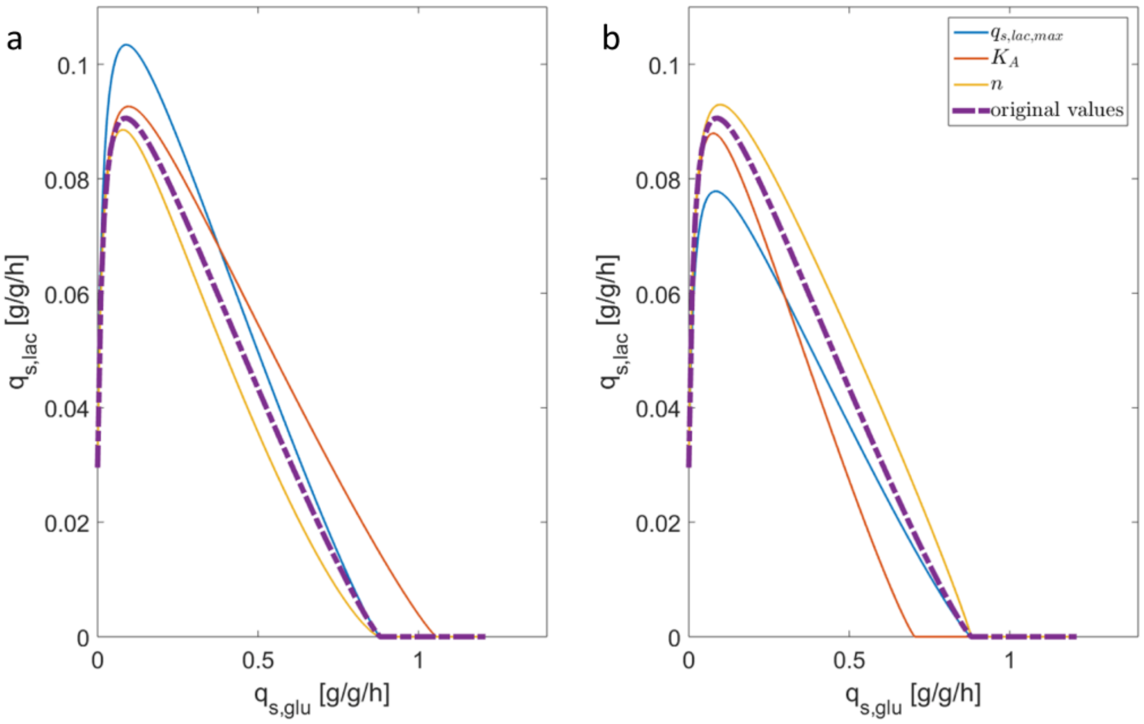

Supplementary Fig. S2 Local sensitivity analysis of the model parameters $q_{s,lac,max}$, K_A and n. The model parameters are (A) decreased or (B) increased by 20% in comparison to the values of the optimal fit
